# Identification and Validation of DLD as a Cuproptosis‐Associated Biomarker in Preeclampsia

**DOI:** 10.1096/fj.202504941R

**Published:** 2026-04-17

**Authors:** Shuisen Zheng, Xiaoling Chen, Wanrou Tang, Danlin Yang, Qing Han

**Affiliations:** ^1^ Department of Obstetrics, Fujian Maternity and Child Health Hospital College of Clinical Medicine for Obstetrics & Gynecology and Pediatrics Fujian Medical University Fuzhou China

**Keywords:** bioinformatics, Cuproptosis, DLD, preeclampsia, WGCNA

## Abstract

Preeclampsia (PE), characterized by new‐onset hypertension and proteinuria after 20 weeks of gestation, presents substantial risks to both mother and fetus. Cuproptosis represents a newly identified form of regulated cell death; its potential association with PE pathogenesis remains unclear. We retrieved the GSE60438 dataset from the GEO database and identified 557 differentially expressed genes (DEGs) associated with PE. Weighted gene co‐expression network analysis (WGCNA) was performed, and by intersecting the DEGs with WGCNA module genes, we obtained 198 candidate PE‐related genes. KEGG and GO enrichment analyses revealed their potential biological functions, with significant enrichment in lipoic acid metabolism, the tricarboxylic acid (TCA) cycle, and components of the mitochondrial matrix. By further intersecting the DEGs, WGCNA module genes, and cuproptosis‐related genes, we identified DLD as the hub cuproptosis‐related gene in PE. GSEA further revealed its involvement in key metabolic pathways. The expression of DLD in PE and normal placental tissues was detected by qRT‐PCR and immunohistochemical staining. Loss or gain‐of‐function tests were performed to assess the effects of DLD on the proliferation, migration, and invasion of HTR‐8/SVneo cells. Concurrently, pyruvate and citrate levels were quantified with commercial kits, and intracellular ultrastructure was examined by transmission electron microscopy. Herein, we detected increased DLD in the PE placental tissues. In vitro studies showed that knockdown of DLD promoted trophoblast proliferation, migration, and invasion; conversely, overexpression of DLD showed the opposite effect. Concurrently, DLD overexpression induced metabolic dysregulation in tricarboxylic acid (TCA) cycle intermediates as well as distinct mitochondrial ultrastructural alterations. Our study demonstrated that DLD, a cuproptosis‐related gene, is significantly upregulated in PE and functionally impairs trophoblast activity, suggesting its pathogenic role may be mediated through cuproptosis.

## Graphical abstract

DLD is identified as a preeclampsia‐associated cuproptosis hub gene by bioinformatics. DLD overexpression inhibits trophoblast proliferation, invasion, and migration, disrupts TCA cycle metabolism, and alters mitochondrial ultrastructure. These findings reveal that DLD may contribute to preeclampsia pathogenesis through cuproptosis.

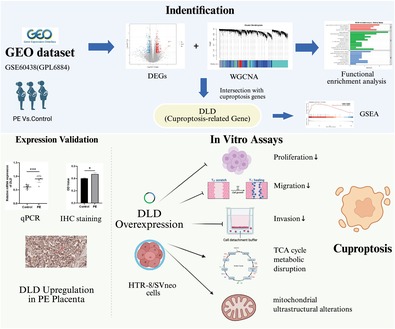

## 1 Introduction

Preeclampsia (PE) is a hypertensive disorder of pregnancy, which affects 2%–8% of pregnancies worldwide and causes significant maternal and perinatal morbidity and mortality [1]. Preeclampsia leads to over 70 000 maternal deaths and 500 000 stillbirths and newborn deaths each year worldwide, posing a major risk to pregnancy outcomes [2]. The pathogenesis of PE involves a complex interaction between inflammation, oxidative stress, vascular injury, and immune dysregulation. This dysregulation contributes to abnormal placental formation and enhanced apoptosis, resulting in insufficient placental invasion, vascular remodeling disorders, and impaired microcirculation [3]. Currently, it is still a challenge to understand the pathogenesis of PE and the mechanisms of PE progression are still elusive.

Copper is an essential trace element in the human body, playing a crucial role as a catalytic cofactor in various biological processes, including mitochondrial respiration and the synthesis of biological compounds [4, 5, 6]. However, copper becomes toxic if its concentration exceeds a certain threshold, inducing a newly discovered form of cell death called cuproptosis. Cuproptosis activation requires functional mitochondrial metabolism and is driven by copper's preferential binding to lipoylated TCA cycle enzymes, most notably dihydrolipoamide S‐acetyltransferase (DLAT) and dihydrolipoamide S‐succinyltransferase (DLST) [7]. The main features of cuproptosis include significant increases in levels of intracellular copper and TCA intermediary metabolites, significant decreases in mitochondrial electron transport chain complex activity, and cellular proliferation, as well as mitochondria morphologic abnormalities and dysfunction [8]. It is known that cuproptosis plays a critical role in the metabolic regulation of tumor cells, such as gastric cancer [9], oral squamous carcinoma [10]. In recent years, some studies have also shown that cuproptosis is closely related to preeclampsia [11]. While the regulatory mechanisms of cuproptosis in PE have remained unclear, the study of its regulatory mechanism is significant.

Bioinformatics is widely used to analyze biological experimental data and reveal the hidden biological meaning of data. Recent developments in gene sequencing have opened new avenues for studying the mechanisms of disease. Tang et al. analyzed GEO datasets and concluded that 5 cuproptosis‐related genes (CRGs) have been closely associated with the pathogenesis of PE [12]. Yu et al. found a potential association between CRGs and the development of PE by using bioinformatics analysis and machine learning [13]. Nevertheless, the correlation between cuproptosis‐related genes and preeclampsia remains to be experimentally validated in cellular models.

In the present study, we screened DLD as a cuproptosis‐associated gene in preeclampsia via bioinformatics analysis. To confirm this result, we verified the expression level of DLD in clinical samples and demonstrated through experiments that DLD has a significant effect on the biological function of placental trophoblasts. This approach enabled us to potentially identify novel strategies for improving adverse maternal and neonatal outcomes in the context of PE.

## 2 Materials and Methods

### 2.1 Data Acquisition and Pre‐Processing

Gene expression data and associated clinical information were retrieved from the Gene Expression Omnibus (GEO) database (http://www.ncbi.nlm.nih.gov/geo). The GSE60438 dataset utilized the GPL6884 data platform for the analysis and included a total of 48 samples (25 PE and 23 normotensive controls) pregnancy [14]. The R language “limma” software package was used to analyze the gene expression difference between PE patients and normal tissues. To improve the robustness of DEG selection, we employed a median absolute deviation (MAD)‐based approach as previously validated in high‐throughput screening studies [15]. Specifically, genes with an absolute log_2_FC exceeding median(|log_2_FC|) + 3 × MAD(|log_2_FC|) and adjust *p*‐value < 0.05 with Benjamini‐Hochberg correction were considered significantly differentially expressed.

We identified copper ion homeostasis‐related gene sets (GOBP_CELLULAR_RESPONSE_TO_COPPER_ION and GOBP_CELLULAR_COPPER_ION_ HOMEOSTASIS) in the molecular signature database (MSigDB; [http://software.broadinstitute.org/gsea/msigdb]) [16]. In addition, previously reported cuproptosis‐related genes that induce cell death were summarized [7]. After eliminating duplicate genes, we identified 55 genes involved in cuproptosis.

### 2.2 Weighed Gene Co‐Expression Network Analysis (WGCNA)

Using the “WGCNA” package (version 4.0.3) and the GSE60438 dataset, we constructed a co‐expression network to identify key modules and hub genes associated with PE. Pearson's correlation coefficients were first calculated to generate an adjacency matrix, which was then transformed into a topological overlap matrix (TOM) after selecting an optimal soft‐thresholding power (*β*) to strengthen biologically meaningful correlations. Hierarchical clustering based on TOM‐based dissimilarity grouped genes with similar expression patterns into distinct modules. Modules significantly correlated with PE (|*r*| > 0.5, *p* < 0.001) were selected based on gene significance, and their constituent genes were retained for downstream analysis.

### 2.3 Functional Enrichment Analysis and GSEA Analyses

The R package “clusterProfiler” was employed to perform Gene Ontology (GO) annotation and Kyoto Encyclopedia of Genes and Genomes (KEGG) enrichment analysis [17]. The GO categories encompassed three sections: biological processes (BP), molecular functions (MF), and cellular components (CC), with a significance threshold set at a *p*‐value < 0.05 for the enrichment pathways. The results were visualized via the R packages “enrichplot” and “ggplot2”. In subsequent analysis, gene set enrichment analysis (GSEA) was conducted on the hub gene using the “clusterProfiler” package, comparing high‐expression versus low‐expression groups.

### 2.4 Placenta Sample

The study population included patients with PE (*n* = 7) and normal pregnancies (*n* = 7). The participants had undergone cesarean section in the Department of Obstetrics, Fujian Maternal and Child Health Hospital from May 2024 to July 2024. The diagnosis of PE was determined according to the guidelines for the diagnosis and treatment of hypertensive disorders in pregnancy (2020) issued by Chinese Medical Association Obstetrics and Gynecology Branch [18]. This study was conducted in accordance with the principles expressed in the Declaration of Helsinki. This study was approved by the Research Medical Ethics Committee of Fujian Maternal and Child Health Hospital (2024KY005).

Several placental tissue samples (0.5 cm × 0.5 cm × 0.5 cm), avoiding visible calcifications and necrotic areas, were obtained from the maternal side of the placenta within 3 min after cesarean section. After removal of maternal blood cells by washing the tissue in sterile phosphate‐buffered saline, it was stored in RNA Later (R0118; Beyotime, Shanghai, China) for later use. The placental tissue for immunohistochemistry was fixed in 4% paraformaldehyde and embedded in paraffin. The remaining samples at −80°C until analysis.

### 2.5 Quantitative Real‐Time Polymerase Chain Reaction (qRT‐PCR)

The total RNA was extracted from issues using RNAiso Plus (Takara, Kyoto, Japan). DLD were detected with NovoScript 1st Strand cDNA Synthesis SuperMix (Novoprotein, Nanjing, China) and NovoStart SYBR qPCR SuperMix Plus (Novoprotein, Nanjing, China). DLD and GAPDH primer sequences (Table 1) were synthesized by Fuzhou Qinke Biological Co. Ltd. The relative expression of target genes was calculated by 2^−ΔΔCt^ method, and each experiment was repeated 3 times.

**TABLE 1:** Primer information.

Target gene	Primer sequence
GAPDH	F: 5′‐GGAGCGAGATCCCTCCAAAAT‐3′
	R: 5′‐GGCTGTTGTCATACTTCTCATGG‐3′
DLD	F: 5′‐GTTGAAGGAATGGCTGGTGG‐3′
	R: 5′‐TGCCCAAGGATCTTCACCATG‐3′

### 2.6 Immunohistochemical Staining (IHC)

Sections were deparaffinized in xylene and rehydrated with a graded series of alcohol. Take an appropriate amount of the antigen retrieval solution diluted to working concentration (1:50) for use prior to immunohistochemical staining. Add an appropriate amount of endogenous peroxidase blocker to inhibit endogenous peroxidase activity. Add 100 μL each of the primary antibody and the enzyme‐labeled goat anti‐mouse/rabbit IgG polymer, then incubate. Tissue sections were stained and counterstained with DAB solution and hematoxylin respectively, dehydrated, and mounted. The staining results were captured using microscopy.

### 2.7 Cell Culture and Cell Transfection

The HTR‐8/SVneo cells used in this study were purchased from Xiamen Immocell Biotechnology Co. Ltd. and cultured in DMEM medium (Procell, Wuhan, China) with 10% fetal bovine serum (ZETA‐LIFE, CA, USA) and 1% Penicillin–Streptomycin Sulfate (Boster, Wuhan, China). The cells were then maintained under humid air containing 5% CO_2_ at 37°C. Cells in the logarithmic growth phase were harvested to prepare a single‐cell suspension and seeded into 6‐well plates at a density of 3 × 10^5^ cells per well. Transfection was performed after cell attachment. For transfection mixture preparation, Solution A (125 μL Opti‐MEM containing 3 μg of OE/sh‐DLD plasmid and 5 μL p3000 enhancer) and Solution B (125 μL Opti‐MEM with 5 μL Lipofectamine 3000) were mixed separately and incubated at room temperature for 5 min. The two solutions were then combined and incubated for an additional 20 min. The cell culture medium was replaced, and the transfection complexes were added dropwise to the cells. After 6–8 h of transfection, the medium was refreshed. Transfection efficiency was assessed 24–48 h post‐transfection.

### 2.8 Cell Counting Kit 8 (CCK8) Assay

Cell proliferation was examined with CCK‐8 (Beyotime, Shanghai, China). First, select cells in optimal growth condition during the logarithmic growth phase to prepare a cell suspension, followed by cell counting. Subsequently, seed the cell suspension at a density of 5 × 10^3^ cells/well in a 96‐well plate, with three replicate wells prepared for each experimental group. The plates are then incubated at 37°C with 5% CO_2_ for designated time intervals (0, 24, 48, and 72 h). Following the incubation period, 10% CCK‐8 reagent is added to each well, and the plates are further incubated for 1 h at 37°C. Finally, the optical density (OD) values of the samples are measured at 450 nm using a microplate reader.

### 2.9 MTT Assay

The assay was performed using an MTT Kit (Beyotime, Shanghai, China) following the manufacturer's instructions. Briefly, cell suspensions with a density of 5 × 10^3^cells/mL were added to 96‐well plates (90 μL per well), with three wells for each group. The cells were incubated at 37°C with 5% CO_2_ for 24 h and then observed under an inverted microscope. Subsequently, 10 μL of MTT solution (0.5% MTT) was added to each well, followed by further incubation for 4 h. The absorbance of each well was measured at 490 nm using a microplate reader.

### 2.10 Scratch Wound Migration Assay

To measure cell migration, an in vitro scratch wound assay was performed. HTR‐8/SVneo cells (5 × 10^5^ cells/well) cultured in 6‐well culture plate for 24 h. Subsequently, cells were scratched with 10 μL pipette tips to create wounds in horizontal as well as in vertical directions parallel to the diameter of the culture plate. Then, the cells were washed with PBS twice and cultured in a serum‐free medium. Images were captured at 0 h and 24 h, and each field of view was imaged three times.

### 2.11 Transwell Migration Assays and Matrigel Invasion Assay

Transwell migration assays and matrigel invasion assay were assessed using cell culture inserts (8.0 μm pore size; LABSELECT, China) without or with Matrigel (MCE, Shanghai, China). After transfection, HTR‐8/SVneo in serum‐free medium were added into the superior compartment of the Transwell. Additionally, the inferior compartment was filled with 600 μL medium containing 10% FBS. The plates were subsequently incubated at 37°C for 24 h, fixed with 4% paraformaldehyde for 20 min, and stained with 0.1% crystal violet for 15 min. Finally, cells in the lower compartment of the chamber were counted under an inverted microscope at a magnification of 10×, and three random fields were selected for cell counting in each chamber.

### 2.12 Determination of Pyruvate and Citrate Levels

Log‐phase cells were trypsinized with 0.25% trypsin and resuspended in serum‐containing medium to prepare a single‐cell suspension. Cells were seeded in 6‐well plates at 3 × 10^5^ cells/well (2 mL/well) and cultured at 37°C with 5% CO_2_ until monolayer formation (triplicate wells). Pyruvate and citrate levels were measured using commercial assay kits (NanJing JianCheng Bioengineering Institute, China) according to manufacturers' protocols (metabolic activity was immediately stopped by ice‐cold PBS washing prior to cell lysis).

### 2.13 Morphological Observation of Trophoblast Cells

Briefly, pelleted cells (approximately 10^7^) were primarily fixed with 2.5% glutaraldehyde for 24 h at 4°C. Dehydration was then carried out using a graded ethanol series (30%, 50%, 70%, 80%, 95%, and 100% twice), with en bloc staining performed in 70% ethanol containing uranyl acetate for 3 h or overnight. The samples were subsequently transitioned to propylene oxide and infiltrated with a mixture of propylene oxide and epoxy resin (1:1) for 2 h, followed by pure epoxy resin for 3 h. Thereafter, samples were embedded in pure epoxy resin. Ultrathin sections (70 nm thick) were cut using a Leica UC‐7 ultramicrotome, collected on copper grids, and counterstained with lead citrate. Finally, images were acquired and analyzed using a transmission electron microscope (JEM1400; JEOL, Tokyo, Japan).

### 2.14 Statistical Analysis

The measurement data are expressed as the mean ± standard deviation (SD). One‐way analysis of variance (ANOVA) and the least significant difference (LSD) test were used to compare the differences among multiple groups. The independent samples *t*‐test was used to compare two groups. When the test level was *α* = 0.05, *p* < *0.05* was considered to indicate a statistically significant difference.

## 3 Results

### 3.1 Identification of DEGs Between PE and Control Samples

**FIGURE 1:**
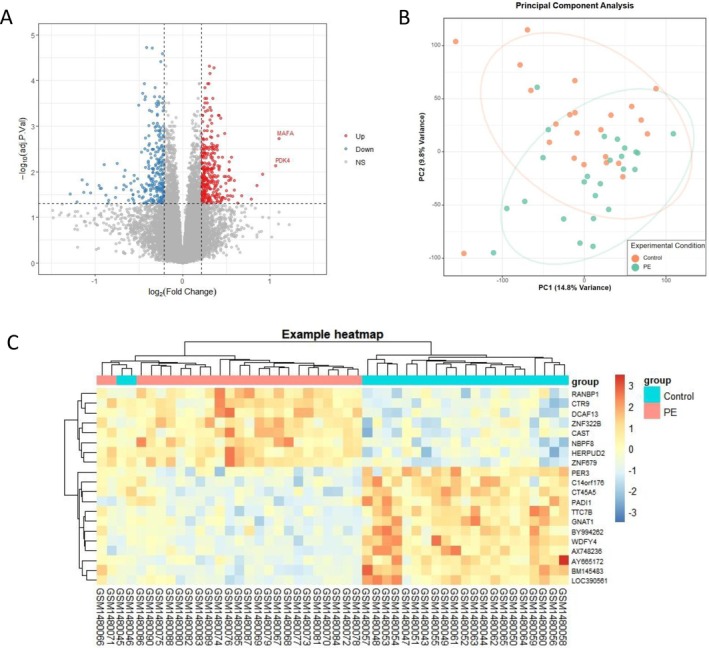
DEGs in PE compared to non‐PE in GSE60438. (A) Volcano plot of DEGs. The upregulated genes are marked in red; the downregulated genes are marked in blue (logFC > 0.2136 and adjust *p*‐value < 0.05). (B) Principal component analysis in GSE60438 datasets. (C) Heatmap shows differential expression of the top 20 genes among 557 PE risk genes among a number of samples.

Gene expression profiles extracted from datasets GSE60438 (GPL6884) were analyzed. After data standardization and differential gene expression analysis, 557 DEGs were found including 302 upregulated and 255 downregulated genes (Figure 1).

### 3.2 Construction and Analysis of Gene Co‐Expression Network

**FIGURE 2:**
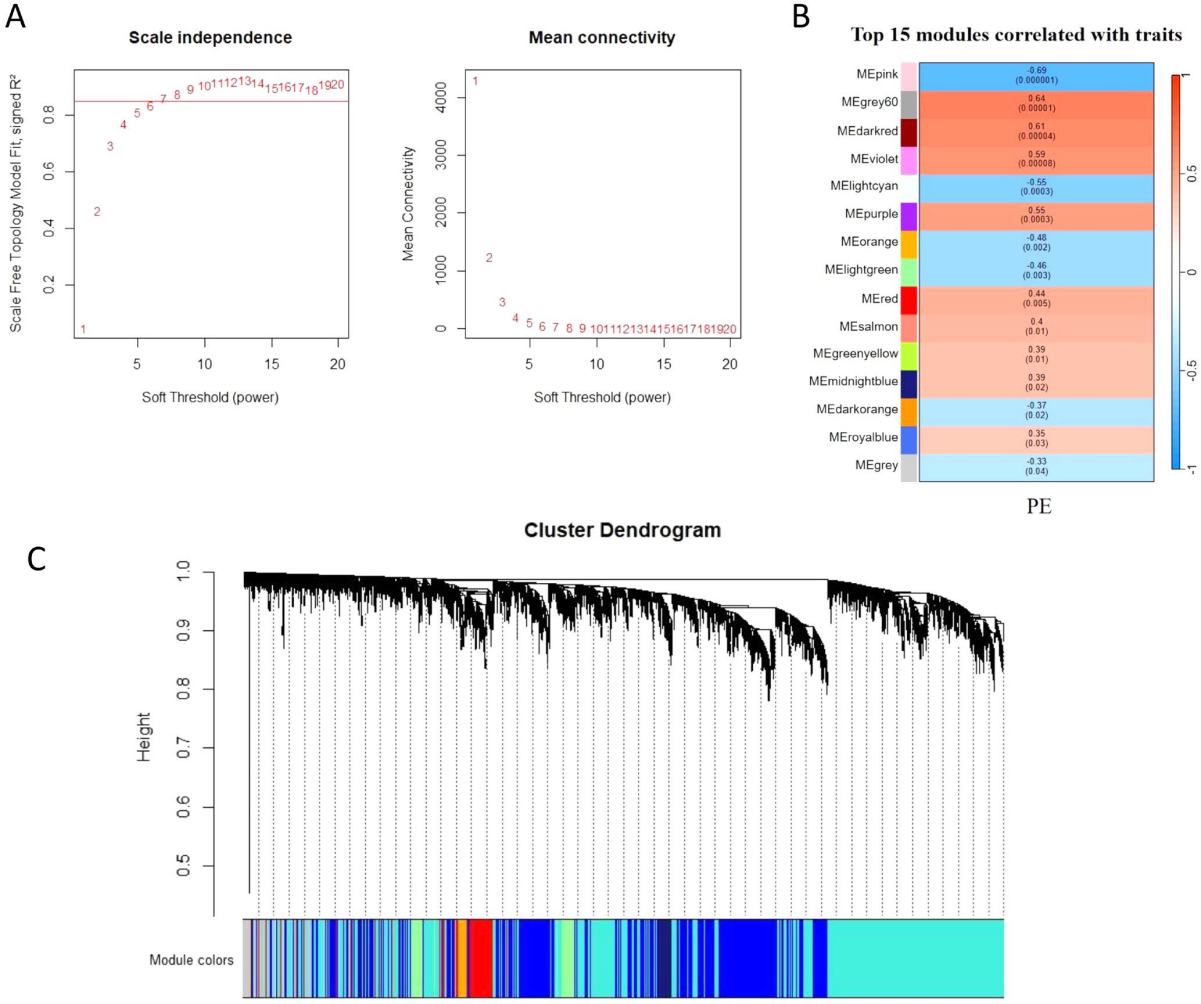
Screening of key genes by WGCNA. (A) The scale independence and the mean connectivity to identify the soft threshold with best performance. (B) The heat map displaying the correlation between the top 15 gene modules and clinical traits. The rows represent gene modules and columns correspond to clinical traits. (C) Hierarchical cluster dendrogram and color‐coding of gene co‐expression modules.

To further precisely excavate the central genes associated with PE, we constructed a gene co‐expression network using the WGCNA algorithm on the dataset of GSE60438. In WGCNA analysis, the soft thresholding power (*β* = 7) was selected to ensure a relatively balanced scale independence and mean connectivity (Figure 2A). In total, 34 gene modules distinct were generated through hierarchical clustering tree, in which each tree branch represented a module, and each leaf constitutes a gene in the dendrogram (Figure 2C). The correlations between modules and PE were computed (Figure 2B). Among these, six modules (pink, grey60, darkred, violet, lightcyan and purple) demonstrated the strongest correlation with PE (|*r*| > 0.5 and *p* < 0.001), and the 2228 genes within these modules were selected for further investigation.

### 3.3 Identification and Functional Enrichment Analysis of Candidate PE‐Related Genes

**FIGURE 3:**
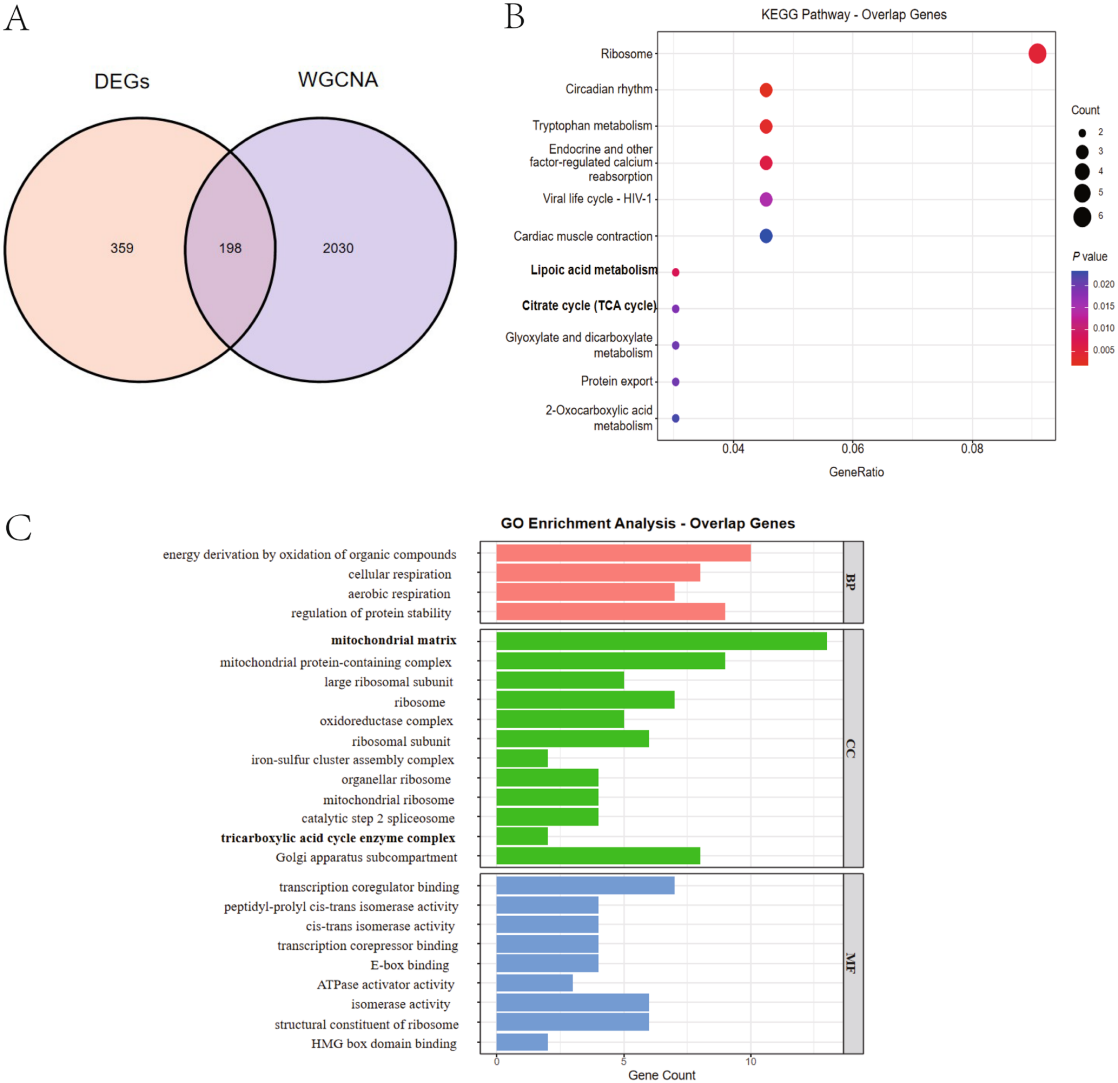
Identification and functional enrichment analysis of candidate signature genes. (A) Venn diagram of DEGs and module genes. (B) KEGG pathway enrichment analysis of candidate signature genes. (C) Gene Ontology (GO) enrichment analysis for candidate signature genes, including biological processes (BP), cellular components (CC), and molecular functions (MF).

To identify candidate PE‐related genes, we first intersected the DEGs with the module genes, yielding 198 candidate PE‐related genes (Figure 3A). These genes were then subjected to functional enrichment analysis to gain insights into their biological functions and potential pathways involved in PE. KEGG analysis highlighted several metabolic pathways, with particular emphasis on lipoic acid metabolism and the tricarboxylic acid (TCA) cycle—two pathways of significant interest to our study. Other enriched pathways included protein processing in the ribosome, circadian rhythm, and tryptophan metabolism (Figure 3B). Cellular component (CC) enrichment was noted in mitochondrial matrix, mitochondrial protein‐containing complex, large ribosomal subunit, and tricarboxylic acid cycle enzyme complex. Biological process (BP) enrichment primarily involved energy derivation by oxidation of organic compounds, cellular respiration, and aerobic respiration. Molecular function (MF) enrichment focused on transcription coregulator binding, peptidyl‐prolyl cis‐trans isomerase activity, and cis‐trans isomerase activity(Figure 3C).

### 3.4 Identification of Hub Cuproptosis‐Related DEGs in PE

**FIGURE 4:**
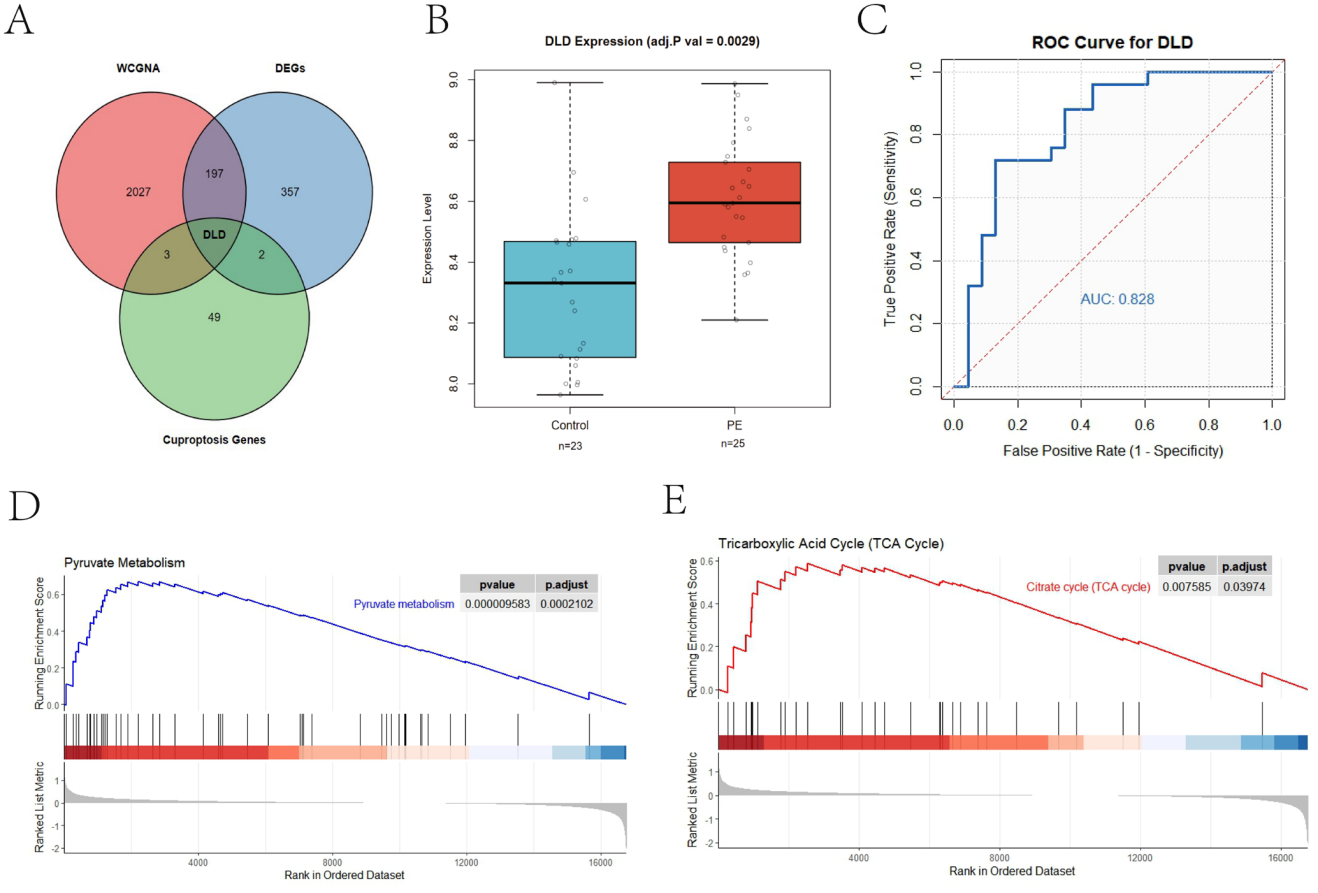
Identification of hub Cuproptosis‐Related DEGs in PE. (A) Venn diagram of cuproptosis‐related genes, DEGs, and module genes. (B) The differential expression of five hub genes between PE and control group in the GSE60438 dataset. (C) The ROC curve of the DLD for diagnosing PE in the GSE60438 dataset. D‐F Identification of DLD‐associated pathways by gene set enrichment analysis (GSEA).

A total of 55 cuproptosis‐related genes (CRGs) were compiled from previously published studies. To identify hub genes associated with both PE and cuproptosis, Venn diagram analysis was performed on the intersection of DEGs, WGCNA module genes, and CRGs. As shown in Figure 4A, DLD was identified as a hub gene common to both PE and cuproptosis. We further examined the expression patterns of DLD between PE and control samples using the GSE60438 dataset, which revealed that DLD was significantly upregulated in PE samples (adjust *P* value = 0.0029; Figure 4B). ROC curve analysis was then performed for DLD, yielding an area under the curve (AUC) of 0.828, indicating its potential as a biomarker to distinguish PE patients from normal pregnant women (Figure 4C). GSEA further demonstrated significant enrichment of pyruvate metabolism (Figure 4D) and the tricarboxylic acid (TCA) cycle (Figure 4F) in the DLD high‐expression group, consistent with its role in cuproptosis, as these are central pathways in this process. These findings suggest that DLD, as a hub gene related to cuproptosis, may serve as a potential predictor for PE.

### 3.5 The Expression of DLD Is Elevated in Placentas of PE

**FIGURE 5:**
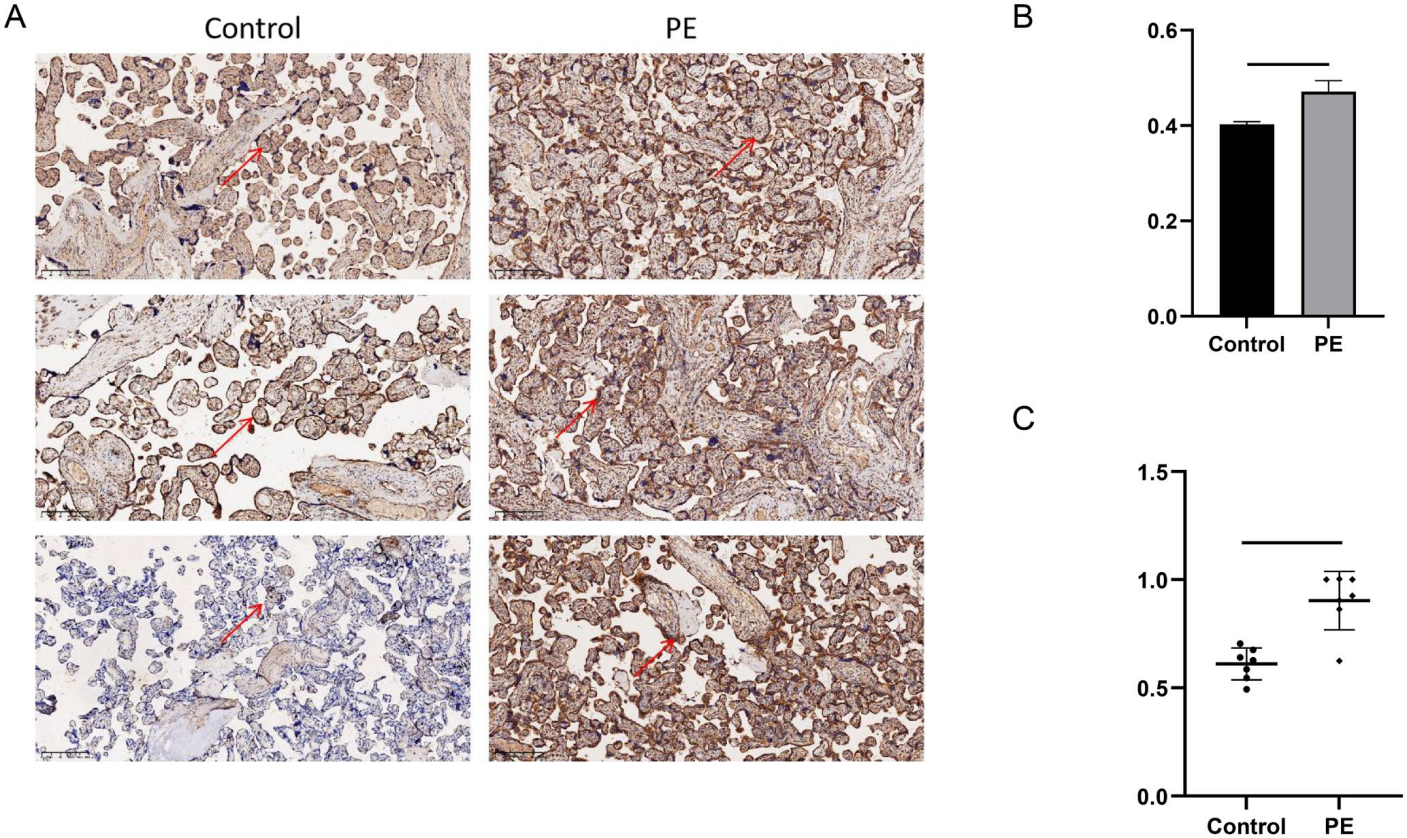
Validation of DLD expression in clinical samples. (A) HE staining of placental tissues from PE and control group. (B) Statistical analysis of DLD expression intensity in IHC specimens using the optical density (OD). (C) qRT‐PCR detection of DLD expression levels in normal placental tissues and PE placental tissues.

To investigate the expression pattern of DLD in PE placental tissue, we compared the levels of DLD in 7 normal placental tissue and 7 PE placental tissue. We examined the protein and mRNA levels of DLD from placental tissues through immunohistochemical staining and qRT‐PCR, respectively in the placental tissues. DLD protein expression was significantly higher in the PE placental tissues than in the normal placental tissues (Figure 5A,B). The same results were observed regarding DLD mRNA expression in placental tissues (Figure 5C).

### 3.6 DLD Inhibited Proliferation, Migration and Invasion of HTR‐8/SVneo Cells

**FIGURE 6:**
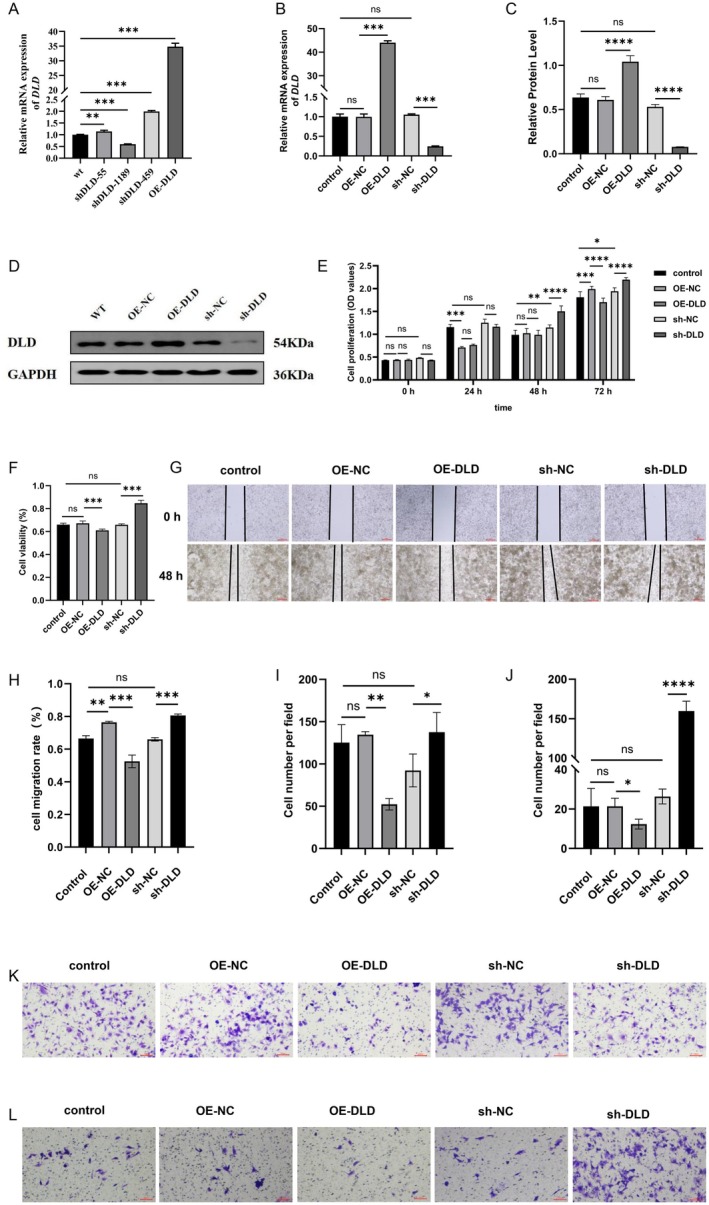
DLD inhibited proliferation, migration and invasion of HTR‐8/SVneo cell. (A) qRT–PCR detected the transfection efficiency of sh‐DLD and OE‐DLD in HTR‐8/SVneo cells. (B) The DLD knockdown and overexpression efficiency in the HTR‐8/SVneo cell was determined by qRT–PCR. (C,D) The DLD knockdown and overexpression efficiency in the HTR‐8/SVneo cell was determined by Western blotting. (E) The proliferation of HTR‐8/SVneo cells after DLD knockdown and overexpression were evaluated by CCK8 assay. (F) The proliferation of HTR‐ 8/SVneo cells after DLD knockdown and overexpression was detected by MTT assay. (G,H) The migratory capacity of HTR‐8/SVneo cells after DLD knockdown and overexpression was determined by wound healing assay. (I, K) The migration of HTR‐8/SVneo cell after DLD knockdown and overexpression were detected via transwell migration assays. (J, L) Cell invasion with underexpression or overexpression of DLD was measured by the Matrigel matrix invasion assay **p < 0.01, ***p < 0.001, ****p < 0.0001.

To further investigate the effects of DLD on the biological behavior of trophoblastic cells, DLD was stably overexpressed or underexpressed in HTR‐8/SVneo cells in vitro. After transfecting HTR‐8/SVneo cells, shDLD‐1189 exhibited optimal efficiency in reducing DLD levels and was used in the following experiments (Figure 6A). The HTR‐8/SVneo cells, when transfected with the DLD‐expressing plasmid, exhibited significantly increased DLD mRNA expression (Figure 6B) and DLD protein expression (Figure 6C,D). The CCK‐8 assay and MTT assay revealed that elevated levels of DLD inhibit cell proliferation, while knockdown of DLD resulted in the promotion of cell proliferation (Figure 6E,F). The results of the wound healing (Figure 6G,H) and transwell migration assays (Figure I and K) demonstrated that increased expression of DLD inhibited the migration of HTR‐8/SVneo cells, whereas its knockdown facilitated this process. We subsequently assessed the effects of DLD inhibition on HTR‐8/SVneo cell invasion. As expected, DLD knockdown significantly enhanced the invasion of HTR‐8/SVneo cells, whereas its overexpression markedly attenuated this process (Figure 6J,L).

### 3.7 DLD Overexpression Mediates TCA Cycle Disruption and Mitochondrial Impairment in Cuproptosis

**FIGURE 7:**
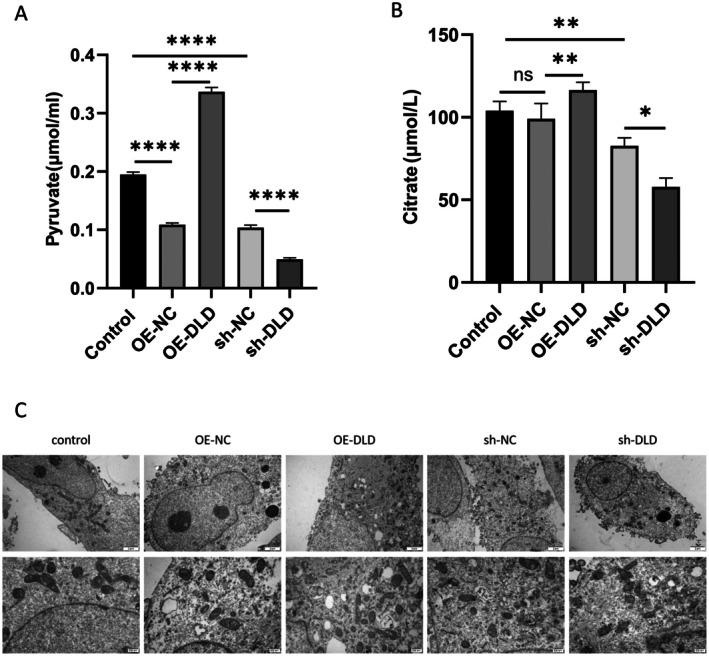
DLD overexpression mediates TCA cycle disruption and mitochondrial impairment in cuproptosis. (A) Pyruvate was detected. (B) Citrate was detected. (C) TEM images showing changes in mitochondria of HTR‐8/SVneo cells with different expression levels of DLD.

The TCA cycle is integral to cuproptosis, and metabolic perturbations within it are a defining feature. Consequently, we measured pyruvate and citrate levels after DLD overexpression and knockdown in HTR‐8/SVneo cells. The results showed that DLD overexpression significantly elevated both metabolites, while conversely, DLD knockdown substantially reduced them (Figure 7A,B). Since cuproptosis induces specific mitochondrial changes, we employed transmission electron microscopy (TEM) to observe the morphological consequences of altering DLD expression in HTR‐8/SVneo cells. Mitochondria in the control group displayed intact membranes and cristae. Similar structures were observed in the overexpression and knockdown control groups (OE‐NC and sh‐NC). However, compared to the OE‐NC group, the OE‐DLD group showed significant cytoplasmic vacuolation, markedly reduced mitochondrial volume, and slightly distorted cristae. In contrast, the sh‐DLD group exhibited no notable structural differences from the sh‐NC control (Figure 7C). Given this striking similarity to the hallmark features of cuproptosis, we propose that DLD overexpression is a potential inducer of this process.

## 4 Discussion

PE is a pregnancy‐associated disorder, which presents as a well‐recognized clinical syndrome characterized by predominantly cardiovascular manifestations attributable to systemic inflammation, endothelial dysfunction, and generalized vasoconstriction resulting in hypertension and multiorgan hypoperfusion. The disorder originates from impaired extravillous trophoblast migration and invasion seen in defective placentation, which leads to defective spiral artery remodeling [19, 20]. Trophoblasts are important functional cells in the placenta essential for maintaining placental function [21]. However, the molecular mechanism underlying the pathogenesis of PE and the regulation of trophoblast behavior remains largely elusive. In our study, KEGG enrichment analysis revealed that PE‐associated genes were predominantly enriched in lipoic acid metabolism and the TCA cycle, two pathways central to cuproptosis. Additionally, GO analysis showed significant enrichment in cellular components including the mitochondrial matrix and the TCA cycle enzyme complex. These results suggest that the pathogenesis of PE may be closely associated with cuproptosis.

Cuproptosis, a recently discovered novel form of programmed cell death mediated by protein lipoylation. The majority of lipoylated proteins are concentrated in the tricarboxylic acid (TCA) cycle. Cells undergoing cuproptosis exhibit heightened TCA cycle activity, which increases the levels of lipoylated TCA enzymes (particularly the pyruvate dehydrogenase complex). This process leads to acute proteotoxic stress and ultimately results in cell death [8]. In 2022, Tsvetkov et al. systematically characterized 10 genes involved in cuproptosis regulation, with functional analyses revealing 7 as activators and 3 as suppressors of this cell death pathway [7]. Following this discovery, genes associated with cuproptosis have emerged as a focal point in biomedical research, as accumulating studies reveal their critical roles in multiple disease pathways [22, 23, 24].

Nevertheless, the potential roles of cuproptosis‐associated genes in the pathogenesis and clinical progression of preeclampsia remain largely unexplored. Thus, we successfully identified the hub cuproptosis‐associated genes in PE in the present study by bioinformatics analysis and machine learning methods. In our study, we identified DLD as a hub cuproptosis‐related DEG in PE through bioinformatics analysis.

DLD, also known as dihydrolipoamide dehydrogenase, is a crucial subunit of the pyruvate dehydrogenase complex (PDC) and plays an important role in the dynamic stability of intracellular copper ions [6, 25]. DLD is mainly localized in the mitochondria and, to a lesser extent, in the nucleus [26]. This enzyme mediates the oxidative decarboxylation of pyruvate to generate acetyl‐CoA [27], an irreversible reaction that supplies essential cofactors for the TCA cycle, thereby serving as a critical metabolic link between glycolysis and oxidative phosphorylation. DLD has emerged as a multifunctional regulator in disease pathophysiology, with demonstrated involvement in acute spinal cord injury [28], lung adenocarcinoma [29], and abdominal aortic aneurysm [30]. However, there has been no study investigating whether DLD can affect PE by inducing cuproptosis. In this study, we screened out DLD as hub CRGs, which may hold significant roles in the progression of PE. To increase the reliability of the results of the bioinformatics analysis, we validated the expression of DLD in samples from PE patients. PE in placenta tissues was significantly greater at both the mRNA and protein levels than it was in normal pregnancy. A loss‐of‐function approach was used to demonstrate that DLD suppresses HTR‐8/SVneo cells' viability, proliferation, invasion, and metastasis in vitro. These findings suggest that DLD has a significant effect on the biological functions of HTR‐8/SVneo cells, which is closely associated with the pathogenesis of PE.

To further explore whether DLD can promote cuproptosis in trophoblast cells, we observed morphological changes in HTR‐8/SVneo cells via transmission electron microscopy. Our study demonstrates that overexpression of DLD induces a series of morphological changes in HTR‐8/SVneo cells, primarily including the appearance of prominent vacuoles, a significant reduction in mitochondrial volume, and mild distortion of mitochondrial cristae. These morphological alterations demonstrate significant similarities with the characteristic ultrastructural features of cuproptosis reported by Zhang et al. [31], particularly in terms of mitochondrial structural compromise. As the core role of maintaining the normal life activities of cells, mitochondria play a pivotal role in regulating cellular energy metabolism, protein quality control, and the regulation of cuproptosis [32, 33]. In line with our GSEA results, DLD overexpression was found to dysregulate key TCA cycle metabolites, exemplified by altered levels of pyruvate and citrate, which functionally confirms the predicted role of DLD in these pathways. These evidences indicate that DLD potentially triggers PE through cuproptosis induction.

There are several limitations. First, the relatively small sample size of our clinical cohort may have introduced bias in case selection. Second, the function of DLD has not been confirmed by in vivo experiments. Third, while DLD showed promising differential expression in our discovery cohort and clinical samples, its expression pattern was inconsistent across publicly available GEO datasets, highlighting the challenges of cross‐dataset validation in heterogeneous conditions such as preeclampsia. Future studies with larger, well‐standardized multi‐center cohorts are warranted to further validate the clinical utility of DLD. Specifically, the involvement of DLD and its associated upstream/downstream pathways in PE pathogenesis requires further investigation through in vivo studies. These important validations will constitute the primary focus of our future research efforts.

## 5 Conclusions

In summary, our study demonstrated that DLD, a cuproptosis‐related gene, is significantly upregulated in PE and functionally impairs trophoblast activity, suggesting its pathogenic role may be mediated through cuproptosis.

## Author Contributions

S.Z. and Q.H. conceived and designed the research. S.Z., X.C., and W.T. acquired data and performed the experiment. S.Z. and D.Y. performed the statistical analysis. Q.H. and S.Z. drafted the manuscript. All authors read and approved the final manuscript.

## Funding

This work was supported by Fujian Provincial Natural Science Foundation of China (2023J011213), Fujian Provincial Health Technology Project (2025QNA056), The Joint Funds for the Innovation of Science and Technology, Fujian Province (2023Y9393, 2025Y9630).

## Disclosure

The authors have nothing to report.

## Ethics Statement

Ethical approval for this study was granted by the ethics committee of Fujian Maternity and Child Health Hospital (2023KY134).

## Consent

Participants gave informed consent to participate in the study before taking part.

## Conflicts of Interest

The authors declare no conflicts of interest.

## Data Availability

The data were anonymized, and no patient information was included to preserve confidentiality. All data used to reach the forementioned conclusions are available for scientific purposes if needed.
